# 

**Published:** 1889-01

**Authors:** 


PAGE.

1889 ... 1
A Conversation with the Grand Lama ... 91
A Materialist Converted ... 60
A Ride ... 168
A Little Poet ... 120
A Victim of Trances ... 50
Apoplexy ... 182
A Test for Tea ... 186
A Cure for Sweating Feet ... 187
A View of the Marriage Question ... 201
A Skeptic's Testimony ... 209
A New Preservative Process ... 252
An Aim at Looking Forward more Wisely .. 221
A Soldier's Life Saved by a Dream ... 87
A Walking Encyclopedia ... 37
An Appreciative Husband ... 115
Amateur Photography ... 35
An Oculist's Test ... 19
A Safe Cordial ... 114
A Good Thing for Boys ... 163
A Loss to Mental Science ... 14
A Remarkable Sensitive ... 33
A Girl's Toilet Articles ... 160
And Now Comes Waterville ... 97
A Paralytic's Recovery ... 30
A Cold and its Treatment ... 280
A Drop of Water ... 285
Buddhism and Theosophy ... 25
Beans ... 259
Bruno ... 288
Convulsions in Children ... 208
Coffee and Typhoid Fever ... 208
"Christian Science" Cures ... 186
Catalepsy ... 182
Consumption, from a Mechanical Standpoint 83
Concerning Criminals ... 62
Celluloise ... 85
Christian Science Outdone ... 112
Care of the Feet ... 9
Curious Cradles ... 40
Cinnamon ... 104
Christian Science. ... 277
Child Whipping ... 280
Disease in the Sewing Machine ... 188
Decoration Day ... 121
Dates ... 83
Dress ... 115
Domestic Science ... 61
Effects of Stimulants on the Voice ... 254
Effect of Occupation, on the face ... 160
Eccentricities in Diet ... 139
Execution by Electricity ... 108
Feeding and Nursing the Sick ... 126
Fruit as Food ... 116
For Dandruff ... 207
Fruit as a Food ... 230
Fingers Before Forks ... 272
Gymnasiums for Girls ... 283
History of a Dewdrop ... 70
How Women Rest ... 103
Hygenic Heresy ... 26
How Celluloid is Made ... 164
Hair Dressing ... 208
Health Resorts ... 145
How Passover Bread is made ... 180
Health without Medicine ... 150
Health without Medicine. ... 174
Health without Medicine ... 197
How Glucose is Made ... 206
How to Live Long ... 9
How New Yorkers are Housed ... 169
Haste and Health ... 154
Human Spontaneous Combust'n ... 250
How Homes are Wrecked ... 203
Haste and Health ... 287
How to Live Long ... 247
Health and Hell, ... 100
Health and Hell ... 122
Health and Hell ... 146
Household ... 21-45-69-94-118-142-166-190-201-213-237-286.
Influence of Music upon Animals ... 38
Indigestion ... 17
In Memoriam ... 72
Infant Feeding ... 258
Insomnia ... 229
Jessie K., on Kissing ... 115
Knitted Dish Cloths ... 116
Looking Forward, No. 1 ... 193
Looking Forward, No. 2 ... 217
Looking Forward, No. 3 ... 241
Looking Forward, No. 4 ... 265
Lincoln's Religion ... 185
Lager Beer as Beverage ... 187
Liquorice Cultivation ... 205
Lard—Its Adulteration, etc ... 110
Looking Forward More Truly ... 221
Meats and their Digestibility ... 251
Modern Diseases ... 232
Music and Lectures ... 251
Magnetic Hygiene ... 56
Monaco ... 58
Modern Indulgences ... 162
Marriage and Birth Statistics ... 274
Measles ... 280
Miscellaneous ... 23-47-95-71-119-143-167-191-214-238-262-287.
Nirvana ... 135
Nature's Transformations ... 258
Not that Kind of a God ... 183
N. Y. Court of Appeals on Adulteration ... 279
Over Their Graves ... 240
Olive Oil in Scarlet Fever ... 259
Our Daughters ... 249
Origin of Strong Liquors ... 109
Only, O Man ... 144
Practical Aids to Beauty ... 250
Pernicious Swindles ... 200
Pure Instinct ... 179
Procrastination ... 48
Precautions in Bathing ... 15
Poveri! Poveris! ... 24
Prevalence of Suicide ... 158
Plea for Prisoners ... 74
Prayer of Socrates ... 79
"Pity 'Tis 'Tis True ... 7
Proper Food for Consumptives ... 273
Psychism ... 276
Paints and Dyes ... 278
Public Hot Baths in China ... 282
Rapidity of the Multiplication of Bacteria. 11
Remarkable Memories ... 11
Rudimentary Organs ... 275
Stick to it, Brethren ... 59
Starved Nerves and Famished Teeth ... 224
Staples Won the Bet ... 228
Searching for a Soul ... 181
Satisfy your Appetite ... 233
Sleeplessness ... 235
Statistics of Breathing ... 176
Suicide ... 170
Save Your Strength ... 185
Some Causes of Cancer ... 18
Science of Psychometry ... 51
San Marino ... 55
Sleep ... 112
Sunflowers and Malaria ... 284
The Death Penalty ... 73
The Human Heart ... 28
The Religion of Buddha ... 138
The Progress of Hindooism in the United States ... 29
The New York World's Fair ... 235
The Future Life ... 225
The Human Body as a Machine ... 65
The Absolute Tests of Death ... 244
Then and Now ... 81
Told by a Snake Charmer ... 226
The Remedies of Nature ... 136
To Cure Drunkenness ... 212
The Consumption of Opium, in China ... 111
The Microbes in Milk and Water ... 207
The Use of Oil to Still the Waves ... 90
The Poison of Tobacco ... 177

PAGE.

The Rattlesnake........................204
The Anæsthetic Revelation.............. 88
The Jak Tree..........................156
The Pasteur Institute.................. 82
The Corset, a Cause of Consumption........162
The Prayer of a Blind Woman............ 43
The True Relations of Filth to Diphtheria 14
Tokology.............................. 281
The Chapin Home........................271
The Raid of the M. D'S ................ 49
The Camphor Tree....................... 67
The Use of Tobacco..................... 12
The Chemistry of Breadmaking........... 31
The Ivory Plant....................... 133
The Vital Functions.................... 16
The Future Life........................ 30
Theosophy.............................. 2
Treatment of Rheumatism................ 15
Treatment of Obesity................... 80
Uses of Cotton Seed....................105
Value of Rest.......................... 19

PAGE.

Ventilation............................161
Vaccination–Its uses and abuses..........130
Valuable Remedies in Diphtheria........ 107
What Drags the Life Out of a Woman....155
What are Tyrotoxicon and Ptomaines?.....255
Why Women get so Short of Breath......184
Weak Hearts............................200
Weak Hearts............................201
Was it a Vision........................179
Why do People Marry?...................253
What are the Thoughts of the Dying......180
Whither are we Drifting................285
Why we are Right Handed................202
Was it Mind-Reading.................... 41
Wonderful Intelligence of a Dog........ 86
Workingmen and Drink...................188
What Shall Appeal to Women............. 7
Winsome Brownie........................ 90
Water in Relation to Obesity...........104
Want of Sleep..........................140
What is Heredity.......................106

### BOOK NOTICES.

We desire to express our thanks for the following complimentary offerings :

INFLATION BY HYDROGEN GAS IN DIAGNOSIS, ETC., by Prof. N. Senn, M. D. Ph. D., of Milwaukee, Wis.

TREATMENT OF DISEASES OF THE NOSE AND PHARYNX, by Prof. W. Cheatham, M. D., Louisville, Ky.

THE PRESIDENT'S ANNUAL ADDRESS, delivered before the American Gynecological Society, 1888.

VOLUME 4, PART 2, OF THE TRANSACTIONS OF THE AMERICAN ŒTOLOGICAL SOCIETY, twenty-first annual meeting. A most complete and valuable compilation, 394 pp., with illustrations.

THE DECEMBER NUMBER OF THE HOME-MAKER, a monthly published at 25 West Twenty-third street, New York City, at $2 per annum, and edited by Marion Harland and associates, is, as its name implies, a journal largely devoted to the home, excellently well presented intellectually, artistically and mechanically.

THE CENTURY FOR JANUARY comes to hand with its usual compliment of excellent and instructive reading, illustrated in the highest style of art. No scholar or would-be scholar can afford to be without it.

SOME OF OUR PETS, with the compliments of the New Year :

*Popular Science News*, *Table Talk*, *Youth's Companion*, *Lend a Hand*, *The Esoteric*, *The Freethinker's Magazine*, *The Children's Friend*, *Babyland*, *Our Little Men and Women*, *The Horticultural Journal*, *Vick's Illustrated Monthly*, *Our Record*, *Good Health*, *Boston Journal of Health*, *La Lotus.*

THE NOVEMBER SATELLITE OF THE ANNUAL OF THE UNIVERSAL MEDICAL SCIENCES, quarterly ; Philadelphia ; F. A. Davis, Publisher, 1231 Filbert street, is at hand. A most valuable contribution to medical and surgical science. No practicing physician can afford to be without it.

